# Level of education and multiple sclerosis risk after adjustment for known risk factors: The EnvIMS study

**DOI:** 10.1177/1352458515579444

**Published:** 2016-01

**Authors:** Kjetil Bjørnevik, Trond Riise, Marianna Cortese, Trygve Holmøy, Margitta T Kampman, Sandra Magalhaes, Kjell-Morten Myhr, Christina Wolfson, Maura Pugliatti

**Affiliations:** Department of Global Public Health and Primary Care, University of Bergen, Norway/The Norwegian Multiple Sclerosis Competence Center, Department of Neurology, Haukeland University Hospital, Norway; Department of Global Public Health and Primary Care, University of Bergen, Norway/The Norwegian Multiple Sclerosis Competence Center, Department of Neurology, Haukeland University Hospital, Norway; The Norwegian Multiple Sclerosis Competence Center, Department of Neurology, Haukeland University Hospital, Norway/The KG Jebsen Centre for MS-Research, Department of Clinical Medicine, University of Bergen, Norway; Institute of Clinical Medicine, Faculty of Medicine, University of Oslo, Norway/Department of Neurology, Akershus University Hospital, Norway; Department of Clinical Neurology, University of Tromsø, Norway/Centre for Clinical Research and Education, University Hospital of North Norway, Norway; Department of Epidemiology and Biostatistics and Occupational Health, McGill University, Canada; The Norwegian Multiple Sclerosis Competence Center, Department of Neurology, Haukeland University Hospital, Norway/The KG Jebsen Centre for MS-Research, Department of Clinical Medicine, University of Bergen, Norway; Department of Epidemiology and Biostatistics and Occupational Health, McGill University, Canada/Research Institute of the McGill University Health Centre, Canada; Department of Global Public Health and Primary Care, University of Bergen, Norway/Department of Clinical and Experimental Medicine, University of Sassari, Italy/Division of Medicine, McGill University, Canada

**Keywords:** Multiple sclerosis, education, socioeconomic status, environmental risk factors

## Abstract

**Background:**

Several recent studies have found a higher risk of multiple sclerosis (MS) among people with a low level of education. This has been suggested to reflect an effect of smoking and lower vitamin D status in the social class associated with lower levels of education.

**Objective:**

The objective of this paper is to investigate the association between level of education and MS risk adjusting for the known risk factors smoking, infectious mononucleosis, indicators of vitamin D levels and body size.

**Methods:**

Within the case-control study on Environmental Factors In MS (EnvIMS), 953 MS patients and 1717 healthy controls from Norway reported educational level and history of exposure to putative environmental risk factors.

**Results:**

Higher level of education were associated with decreased MS risk (*p* trend = 0.001) with an OR of 0.53 (95% CI 0.41–0.68) when comparing those with the highest and lowest level of education. This association was only moderately reduced after adjusting for known risk factors (OR 0.61, 95% CI 0.44–0.83). The estimates remained similar when cases with disease onset before age 28 were excluded.

**Conclusion:**

These findings suggest that factors related to lower socioeconomic status other than established risk factors are associated with MS risk.

## Introduction

Multiple sclerosis (MS) is a demyelinating disease of the central nervous system whose etiology is unknown. While the evidence is strong that both genetic and environmental factors contribute to the risk of the disease,^1^ currently known risk factors are not likely to fully explain individual disease risk. This suggests that yet unknown risk factors are important in the etiology of the disease.

Socioeconomic status (SES) can predict the risk of a range of diseases, including cardiovascular disease,^2^ type 2 diabetes^3^ and certain types of cancer.^4^ Previous research on SES and MS has, however, produced conflicting results.^5^ Both higher^6^ and lower^7,8^ levels of SES have been associated with an increased disease risk. Although there may be geographical differences in how SES affects MS risk, some of the conflicting results may also be explained by methodological limitations, such as lack of adjustment for known risk factors. While a recent study reported an association between lower levels of SES and a higher MS risk that persisted after adjusting for several known risk factors,^9^ it was not able to account for measures of vitamin D.

**Figure 1.**
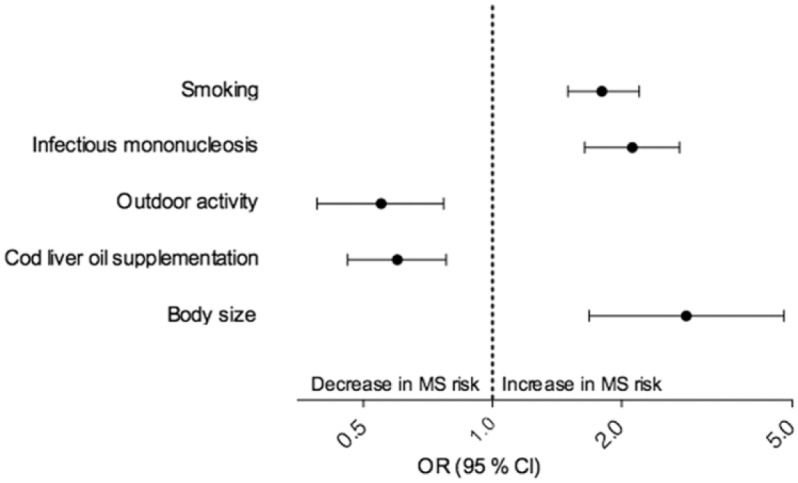
Risk factors in EnvIMS. OR of MS risk for risk factors reported in previous studies using EnvIMS data. The effect estimates reflect the difference between “ever-smoker” and “never-smoker,” “infectious mononucleosis” and “no infectious mononucleosis,” “outdoors most of the time” and “no outdoor activity,” “highest consumption of cod liver oil” and “no consumption of cod liver oil” and “largest body size” and “smallest body size.” EnvIMS: Environmental Factors in Multiple Sclerosis; OR: odds ratio; MS: multiple sclerosis; CI: confidence intervals.

In this study, we examined the association between participant education, a valid indicator of SES,^10^ and MS in the setting of a large case-control study where findings of the environmental risk factors most consistently associated with MS have been reproduced (Figure 1).^11–15^ Furthermore, we wanted to see to what degree these known risk factors could explain any association between education and MS.

## Methods

### Study design

This study is a part of the international multicentric case-control study of Environmental Factors in Multiple Sclerosis (EnvIMS). The EnvIMS study was carried out in well-defined geographic areas in Europe (Norway, Italy, Serbia and Sweden) and in Canada. It aimed at examining the effect of self-reported exposure to environmental risk factors in MS prior to disease onset and to disclose possible variations in risk between distinct populations using a common methodology. The study design and methodology have been reported elsewhere.^16^

### Study area and population

The current study used the Norwegian EnvIMS data. Norway is situated between 58 and 71 degrees northern latitude and has an MS prevalence among the highest reported in the world, ranging from 106 to 245 per 100,000 in surveys from different counties.^17^ Cases were selected from the Norwegian MS-registry and biobank (Haukeland University Hospital, Bergen), which recruits patients from the whole country.^18^ They had been diagnosed according to the McDonald criteria with a clinical onset within 10 years prior to data collection (i.e. 1999–2008).^19^ Four times as many age and sex frequency-matched controls were randomly selected from the population-based National Registry in Norway. Only participants aged 18 years or older at the time of selection were included in the study.

### Exposure

Exposure information was collected through a novel, self-administered questionnaire (EnvIMS-Q) that had been tested for reliability, cross-cultural validity and perceived difficulty of completion.^16^ In Norway, the level of education was reported on a five-point scale including “7 years or less” (elementary school), “8–10 years” (middle school), “11–13 years” (high school), “14 years or more” (college/university) and “I do not know.” Covariates reflecting risk factors most commonly associated with MS risk included history of smoking, history of infectious mononucleosis, proxies of vitamin D levels (outdoor activity and sun exposure, vitamin D supplementation, fatty fish intake) and body size.

Smoking habits were reported as “ever” and “never” smoker, cigarettes/day (1–4, 5–10, 11–20 and 20+) in specific age periods (11–15, 16–20, 21–25 and 26–30), age at smoking initiation and total years of smoking. Those who started smoking after disease onset were classified as “never” smokers. History of infectious mononucleosis was reported as “yes,” “no” and “I do not remember.” Frequency of outdoor activity, a proxy for sun exposure and vitamin D levels, was reported for both winter and summer in the specific age periods 0–6, 7–12, 13–15, 16–18, 19–24 and 25–30. It was reported as “virtually all the time,” “quite often,” “reasonably often” and “not that often.” The frequency of use of vitamin D-containing supplements (e.g. cod liver oil) in the age period 13–19 was reported as “never/seldom,” “1–3 times/month,” “1 time/week,” “2–3 times/week,” “4–6 times/week” and “7+ times/week.” Information on intake of specific fish species (herring, mackerel, halibut, flounder, salmon and trout) was reported as “never/seldom,” “1 time/month,” “2–3 times/month,” “1 time/week,” “2 times/week” and “3 and more times/week.” A figure rating scale consisting of body sketches, which reflect individuals’ body mass index (BMI),^20^ was used as an estimate for BMI. This scale was used for the specific ages 5, 10, 15, 20, 25 and 30.

### Statistical analysis

The association between disease and exposure was estimated as odds ratios (OR) with 95% confidence intervals (95% CIs) using logistic regression. A new variable for education was created where the two lowest categories in the education variable (“7 years or less” and “8–10 years”) were merged and given the value 1, 11–13 was given the value 2 and 14 or more was given the value 3. The two categories reflecting the shortest education were merged as they constitute the compulsory years of education in the Norwegian education system and very few participants have only seven years of education. The level of education was then treated as a categorical variable using category 1 as the reference category. Information on intake of each fatty fish was combined into one variable accounting for the intake frequency of any fatty fish per time unit with the categories “never,” “1–2 times/month,” “3–4 times/month,” “5–6 times/month” and “7 or more times/month.” Smoking, infectious mononucleosis, outdoor activity, vitamin D supplementation, fatty fish intake and self-reported body size were assumed to be possible confounding or mediating factors and were introduced one by one into a multivariable model. For exposures with several age categories, the age category most relevant to MS according to previous findings in the EnvIMS study and other studies were selected. Specifically, this included age 16–18 for outdoors activity and body size at age 20. Cox-Snell *R*^2^ was used to estimate the proportion of variance accounted for by the fully adjusted model.

We compared the results in early and late study responders, a suggested method for evaluating selection bias due to non-response.^21^ Early responders were defined as those who answered after first contact, while late responders were defined as those who answered after a reminder.

All controls were randomly assigned an index age based on the distribution of age of disease onset in the cases. Events or reported behavior occurring after the age of onset/index age were not considered as relevant exposure. The analyses were repeated including only cases with age of disease onset after 28 years, to account for possible reverse causality due to disease-related processes affecting the possibility to take part in higher education. All analyses were adjusted for age and sex.

The statistical analyses were performed in IBM SPSS Statistics for Macintosh, version 22, Armonk, NY: IBM Corp.

### Protocol approvals and patient consents

EnvIMS-Q is an anonymous postal questionnaire with an identical format for both cases and controls. A cover letter with the study aims and relevance, the request and instructions for participation and the investigators’ contact information were enclosed with the questionnaire. Return of the questionnaire was considered as evidence of consent.

The Norwegian component of the EnvIMS study received ethics approval by the Regional Ethical Committee for Medical and Health Research for Western Norway (n. 11, 18.12.2008).

## Results

A total of 1368 eligible cases and 4728 controls were invited to participate and the response rates were 69.7% and 36.3%, respectively.

There were statistically significant differences in the distribution of baseline characteristics according to levels of education among participants in EnvIMS (Table 1). Those with lower levels of education were more likely to frequently spend time outdoors, consume fatty fish during adolescence and to be an ever-smoker. Infectious mononucleosis and cod liver oil supplementation during adolescence were more frequently reported among participants with higher education.

**Table 1.** Characteristics of participants and distributions of risk factors according to level of education^a^.

Characteristics	Level of education				
	Elementary school (7 years or less)	Middle school (9–10 years)	High school (11–13 years)	College/University (14 years or more)	*p*
*N*	64	290	986	1292	
Year of birth (SD)	1946 (8.4)	1958 (10.6)	1964 (10.4)	1965 (10.0)	<0.001
Sex (female:male)	2.6:1	1.8:1	2.4:1	2.9:1	0.006
Tobacco smoke (ever), %	70.5	77.0	60.7	46.1	<0.001
Infectious mononucleosis (ever), %	1.7	7.0	10.6	13.4	0.001
Outdoor activity, %					
Not that often	2.2	5.3	5.4	5.7	0.005
Reasonably often	43.5	37.9	43.8	43.8	
Quite often	39.1	43.2	40.6	44.0	
Virtually all the time	15.2	13.6	10.2	6.5	
Cod liver oil supplementation, %					
Never	65.5	65.0	66.4	59.0	0.029
1 time/week or less	6.9	5.9	7.3	8.2	
2–3 times/week or more	27.6	29.1	26.2	32.7	
Fatty fish consumption, %					
Never/seldom	12.9	12.7	14.6	9.9	<0.001
1–3 times/month	16.1	37.1	37.4	40.0	
Weekly	71.0	50.2	48.0	50.0	
Estimated BMI (kg/m^2^) at age 20, %					
<20	50.0	41.8	37.4	35.7	0.033
20–25	33.9	44.8	46.2	50.8	
26–30	12.5	12.3	14.9	12.8	
>30	3.6	1.1	1.5	0.7	

a χ^2^ statistics and analysis of variance (ANOVA) were used to compare categorical and continuous characteristics, respectively. BMI: body mass index.

We found a statistically significant association between level of education and risk of MS. Participants with a higher level of education had a lower MS risk compared to those with lower levels of education (OR 0.53, 95% CI: 0.41–0.68). Table 2 gives the results for univariate analyses for each variable separately, a model including education and smoking and the final model including all considered confounders. Changes in the effect estimate of education were seen only when adjusting for smoking, although the changes were small and the estimate remained highly significant (OR 0.59, 95% CI: 0.45–0.77). Further adjustment for infectious mononucleosis, frequency of outdoor activity, cod liver oil supplementation, fatty fish intake and body size did not markedly change the effect estimate. A statistically significant *p* trend for education was observed both for the crude (*p* < 0.001 for trend) and adjusted analyses (*p* = 0.002 for trend). For the fully adjusted model, Cox-Snell *R*^2^ was 0.055.

**Table 2.** The association between level of education and MS risk adjusting for possible confounders.

	Model 1^a^	Model 2^b^	Model 3^c^
	*P*^d^	*P*^d^	*P*^d^
	OR (95% CI)	OR (95% CI)	OR (95% CI)
**Education**	< 0.001	<0.001	0.002
Compulsory	1	1	1
Secondary	**0.76** (0.59–0.98)	**0.79** (0.60–1.03)	**0.81** (0.59–1.11)
Tertiary	**0.53** (0.41–0.68)	**0.59** (0.45–0.77)	**0.61** (0.44–0.83)
**Smoking**	<0.001	<0.001	<0.001
No	1	1	1
Yes	**1.87** (1.57–2.22)	**1.75** (1.47–2.09)	**1.70** (1.40–2.08)
**Infectious mononucleosis**	<0.001		<0.001
No	1		1
Yes	**2.11** (1.64–2.73)		**2.29** (1.73–3.04)
**Outdoor activity**	0.001		0.042
Max. versus min. exposure	**0.55** (0.39–0.77)		**0.65** (0.43–0.98)
**Cod liver oil**	0.001		0.032
Max. versus min. exposure	**0.67** (0.54–0.84)		**0.75** (0.58–0.98)
**Fatty fish consumption**	0.011		0.091
Max. versus min. exposure	**0.70** (0.54–0.92)		**0.76** (0.55–1.04)
**Body size at age 20**	<0.001		0.022
Max. versus min.	**2.44** (1.50–3.99)		**1.92** (1.10–3.37)

a Univariate model of each variable separately, adjusted for age and sex.

b Multivariable model including education, smoking, age and sex.

c Multivariable model including education, smoking, infectious mononucleosis, outdoor activity, cod liver oil supplementation, fatty fish consumption, body size, age and sex.

d Level of significance when comparing the highest and lowest level of exposure.

OR: odds ratio; CI: confidence interval.

A higher proportion of the controls compared to cases (25.5% vs 15.6%) were late responders. The effect estimate of education on MS risk in the fully adjusted model was slightly stronger among late responders (OR 0.54, 95% CI: 0.26–1.11) than in early responders (OR 0.62, 95% CI: 0.43–0.89), but there was no significant interaction (*p* = 0.48).

The analyses were repeated including only cases and controls with age of disease onset or index age of 28 or older. Both the crude (OR 0.53, 95% CI: 0.41–0.69) and adjusted effect estimate (OR 0.60, 95% CI: 0.43–0.84) remained similar. Furthermore, adjusting for the educational and ethnic background of the parents did not markedly change the crude (OR 0.52, 95% CI: 0.40–0.68) or the adjusted effect estimates (OR 0.63, 95% CI: 0.45–0.87).

## Discussion

We observed that a higher level of education was associated with lower MS risk in the Norwegian population of the EnvIMS study. This association remained similar after we adjusted for the environmental factors most consistently associated with MS, which may imply that education could be a marker for unknown exposures that are important for the etiology of the disease.

Our findings are consistent with observations in several recent studies. A large, prospective, registry-based study with approximately 400,000 participants from the same source population as our study reported an association in the same direction with similar effect estimates.^7^ Further, a recent study reported that lower level of both parental and the participants’ own education was associated with a higher MS risk after adjusting for several environmental and genetic risk factors.^9^ Due to little variation in proxies of vitamin D status (e.g. vitamin D supplementation) they were unable to adjust for these in the analyses. Lastly, a large Danish cohort study reported a lower MS risk among children of mothers with higher education.^8^

Some earlier studies have shown the opposite or no association between education and MS risk.^6, 22^ This is in agreement with some of the earliest studies on SES and MS, where several studies reported that there was a higher ratio of MS patients among professional workers, and thereby people with higher education, than in unskilled workers.^6,22^ The hygiene hypothesis, where the overall function of the immune system is altered due to lower exposure to pathogens early in life, has often been cited to explain findings where higher SES is associated with higher MS risk.^8^

These conflicting results may be due to methodological limitations, but also to a change in the distribution of risk factors for MS. The habit of smoking, an established risk factor for MS,^23^ varies with time and place. While the global smoking prevalence continues to decline,^24^ the socioeconomic differences in smoking prevalence are increasing due to a smaller decline in smoking rates among groups with lower levels of SES.^25,26^ Similarly, the distribution of other measured and unmeasured factors related to MS risk may also have changed, which could explain some of the heterogeneity observed. In the present study we observed that smoking could account for some of the association between SES and MS risk, but that other relevant exposures did not change the effect estimates. This is consistent with the no clear-cut association between these factors and education found in this Norwegian study population.

These findings are therefore likely related to other risk factors associated with SES. Lower levels of SES have been associated with higher levels of urinary overnight cortisol^27^ and inflammatory markers in serum,^28^ suggesting that exposures related to SES may alter the hypothalamic-pituitary-adrenal (HPA) axis and allostatic load over time. Although research on stress and MS risk is not consistent, some studies report an association between the two.^29^ Further, SES is associated with marked differences in dietary patterns.^30^ Components in the diet that are not depicted in more general dietary patterns, which were not associated with MS risk,^31^ could be important. Recently, sodium intake^32^ and the composition of the gut microbiota^33^ have been proposed as potential risk factors for MS. Although this is mainly based on animal research, these factors could both be associated with SES and provide biologically plausible pathways for a subsequently altered MS risk.

This study has some limitations. First, studies with non-responders are prone to selection bias. The probability of responding to a questionnaire may be related to the education of those invited^34^ and could lead to a selection of participants with higher levels of education into the study, both among cases and controls. Still, less-motivated controls may be more sensitive to this selection than more-motivated patients. Thus, selection bias could occur if controls have lower response rates compared to cases, as we observed in our study. The observed differences in our study could explain some of the results. We compared the effect estimates in early and late responders, as late responders may be more similar to non-responders.^**21**^ No significant differences were found, and the effect estimates were actually slightly stronger among late responders. Further, considering the similarities of the results in our study and the large prospective study with participants from the same source population, it is unlikely that our results can be fully explained by selection bias.

A second limitation is that case-control studies are also prone to recall bias as the participants are asked to retrospectively recall prior exposure information. The recollection of level of education is, however, less likely to be affected after disease onset. We observed age-specific differences in the reported exposure of several relevant mediators and confounders.^11,15^ It is unlikely that a differential recall between cases and controls should vary according to age periods if they were due to recall bias. Moreover, although we had several measures for various risk factors, there may still be residual confounding that we are not able to account for.

In conclusion, in this large population-based study we observed that a higher level of education was associated with lower MS risk that could not be fully explained by currently established risk factors including smoking, vitamin D and infectious mononucleosis. This strongly suggests the presence of additional environmental risk factors in the etiology of MS.

## References

[1] Ascherio A Munger KL Lunemann JD The initiation and prevention of multiple sclerosis. Nat Rev Neurol 2012; 8: 602–612. 23045241 10.1038/nrneurol.2012.198 PMC4467212

[2] Fiscella K Tancredi D Franks P Adding socioeconomic status to Framingham scoring to reduce disparities in coronary risk assessment. Am Heart J 2009; 157: 988–994. 19464408 10.1016/j.ahj.2009.03.019

[3] Lee TC Glynn RJ Peña JM Socioeconomic status and incident type 2 diabetes mellitus: Data from the Women’s Health Study. PloS One 2011; 6: e27670. 22194788 10.1371/journal.pone.0027670 PMC3237410

[4] Mouw T Koster A Wright ME Education and risk of cancer in a large cohort of men and women in the United States. PloS One 2008; 3: e3639. 18982064 10.1371/journal.pone.0003639 PMC2572908

[5] Goulden R Ibrahim T Wolfson C Is high socioeconomic status a risk factor for multiple sclerosis? A systematic review. Eur J Neurol. Epub ahead of print 5 11 2014 DOI: 10.1111/ene.12586. 25370720

[6] Kurtzke JF Page WF Epidemiology of multiple sclerosis in US veterans: VII. Risk factors for MS. Neurology 1997; 48: 204–213. 9008519 10.1212/wnl.48.1.204

[7] Riise T Kirkeleit J Aarseth JH Risk of MS is not associated with exposure to crude oil, but increases with low level of education. Mult Scler 2011; 17: 780–787. 21343231 10.1177/1352458510397686

[8] Nielsen NM Jørgensen KT Bager P Socioeconomic factors in childhood and the risk of multiple sclerosis. Am J Epidemiol 2013; 177: 1289–1295. 23660795 10.1093/aje/kws350

[9] Briggs FB Acuña BS Shen L Adverse socioeconomic position during the life course is associated with multiple sclerosis. J Epidemiol Community Health 2014; 68: 622–629. 24577137 10.1136/jech-2013-203184

[10] Shavers VL Measurement of socioeconomic status in health disparities research. J Natl Med Assoc 2007; 99: 1013–1023. 17913111 PMC2575866

[11] Bjørnevik K Riise T Casetta I Sun exposure and multiple sclerosis risk in Norway and Italy: The EnvIMS study. Mult Scler 2014;20: 1042–1049. 24414538 10.1177/1352458513513968

[12] Lossius A Riise T Pugliatti M Season of infectious mononucleosis and risk of multiple sclerosis at different latitudes; the EnvIMS Study. Mult Scler 2014; 20: 669–674. 24072723 10.1177/1352458513505693

[13] Wesnes K Riise T Casetta I Body size and the risk of multiple sclerosis in Norway and Italy: The EnvIMS study. Mult Scler 2015; 21: 388–395. 25182290 10.1177/1352458514546785

[14] Riise T Pugliatti M Casetta I Negative interaction between smoking and infectious mononucleosis in the risk of MS. ECTRIMS 5th Joint Triennial Congress, Amsterdam Mult Scler 2011; 17: S9-S52, abstract no. 131.22069791

[15] Cortese M Riise T Bjørnevik K Timing of cod liver oil use as a vitamin D source and multiple sclerosis risk in Norway: The EnvIMS study. 2014 Joint ACTRIMS-ECTRIMS Meeting (MSBoston 2014) MS Journal Online: Poster Session 1, P331. Mult Scler 2014; 20: 67–284.25205048

[16] Pugliatti M Casetta I Drulovic J A questionnaire for multinational case-control studies of environmental risk factors in multiple sclerosis (EnvIMS-Q). Acta Neurol Scand Suppl 2012: 43–50. 23278656 10.1111/ane.12032

[17] Midgard R Incidence and prevalence of multiple sclerosis in Norway. Acta Neurol Scand Suppl 2012: 36–42. 23278655 10.1111/ane.12025

[18] Myhr KM Grytten N Aarseth JH The Norwegian Multiple Sclerosis Registry and Biobank. Acta Neurol Scand Suppl 2012: 20–23. 23278652 10.1111/ane.12030

[19] Polman CH Reingold SC Edan G Diagnostic criteria for multiple sclerosis: 2005 revisions to the “McDonald Criteria”. Ann Neurol 2005; 58: 840–846. 16283615 10.1002/ana.20703

[20] Bulik CM Wade TD Heath AC Relating body mass index to figural stimuli: Population-based normative data for Caucasians. Int J Obes Relat Metab Disord 2001; 25: 1517–1524. 11673775 10.1038/sj.ijo.0801742

[21] Lindner JR Murphy TH Briers GE Handling nonresponse in social science research. J Agri Educ 2001; 42: 43–53.

[22] Kotzamani D Panou T Mastorodemos V Rising incidence of multiple sclerosis in females associated with urbanization. Neurology 2012; 78: 1728–1735. 22592376 10.1212/WNL.0b013e31825830a9

[23] Riise T Nortvedt MW Ascherio A Smoking is a risk factor for multiple sclerosis. Neurology 2003; 61: 1122–1124. 14581676 10.1212/01.wnl.0000081305.66687.d2

[24] Ng M Freeman MK Fleming TD Smoking prevalence and cigarette consumption in 187 countries, 1980–2012. JAMA 2014; 311: 183–192. 24399557 10.1001/jama.2013.284692

[25] Corsi DJ Boyle MH Lear SA Trends in smoking in Canada from 1950 to 2011: Progression of the tobacco epidemic according to socioeconomic status and geography. Cancer Causes Control 2014; 25: 45–57. 24158778 10.1007/s10552-013-0307-9

[26] Garrett BE Dube SR Trosclair A Cigarette smoking—United States, 1965–2008. MMWR Surveill Summ 2011; 60 (Suppl): 109–113. 21430635

[27] Evans GW Kim P Childhood poverty and health: Cumulative risk exposure and stress dysregulation. Psychol Sci 2007; 18: 953–957. 17958708 10.1111/j.1467-9280.2007.02008.x

[28] Loucks EB Pilote L Lynch JW Life course socioeconomic position is associated with inflammatory markers: The Framingham Offspring Study. Soc Sci Med 2010; 71: 187–195. 20430502 10.1016/j.socscimed.2010.03.012 PMC2895737

[29] Artemiadis AK Anagnostouli MC Alexopoulos EC Stress as a risk factor for multiple sclerosis onset or relapse: A systematic review. Neuroepidemiology 2011; 36: 109–120. 21335982 10.1159/000323953

[30] Wang DD Leung CW Li Y Trends in dietary quality among adults in the United States, 1999 through 2010. JAMA Intern Med 2014; 174: 1587–1595. 25179639 10.1001/jamainternmed.2014.3422 PMC5924699

[31] Rotstein D Chiuve S Chitnis T Dietary patterns not associated with the risk of multiple sclerosis. 2014 Joint ACTRIMS-ECTRIMS Meeting (MSBoston 2014): Oral presentations, PS5.3. Mult Scler 2014; 20: 14–66.25205047

[32] Kleinewietfeld M Manzel A Titze J Sodium chloride drives autoimmune disease by the induction of pathogenic TH17 cells. Nature 2013; 496: 518–522. 23467095 10.1038/nature11868 PMC3746493

[33] Berer K Mues M Koutrolos M Commensal microbiota and myelin autoantigen cooperate to trigger autoimmune demyelination. Nature 2011; 479: 538–541. 22031325 10.1038/nature10554

[34] Sonne-Holm S Sørensen TI Jensen G Influence of fatness, intelligence, education and sociodemographic factors on response rate in a health survey. J Epidemiol Community Health 1989; 43: 369–374. 2614328 10.1136/jech.43.4.369 PMC1052876

